# Transient Mood Elevation Without a Manic Switch: Goldenseal-Augmented Oral Glutamatergic Regimen in Bipolar Depression With Comorbid Obsessive-Compulsive Disorder (OCD)

**DOI:** 10.7759/cureus.104209

**Published:** 2026-02-24

**Authors:** Ngo Cheung

**Affiliations:** 1 Psychiatry, Cheung Ngo Medical Limited, Hong Kong, HKG

**Keywords:** bipolar, bipolar disorders, cheung regimen, glutamatergic, goldenseal

## Abstract

Treating bipolar depression that comes with obsessive, looping thoughts can be tricky because standard antidepressants often help only a little and can sometimes push the mood too high. In this report, we followed a 32-year-old man whose low mood and health-related ruminations lingered despite mood stabilisers (valproate, low-dose aripiprazole, pregabalin, and Deanxit). Hoping to copy some of ketamine's brain-circuit effects, he tried an over-the-counter mix of dextromethorphan, piracetam, and L-glutamine; the result was modest at best. Ten days after he added 600 mg of the herbal CYP2D6 inhibitor goldenseal, thought to slow the breakdown of dextromethorphan, he reported a clear lift in energy, mood, and work performance, with no signs of hypomania; the improvement faded when he stopped the herb and returned when he restarted it, and he had no side effects. Although this single experience cannot show cause and effect, it hints that short courses of a well-standardised botanical CYP2D6 inhibitor might prolong the benefit some patients get from oral dextromethorphan-based combinations, an idea that would need careful study, including drug-level monitoring and checks for mood switches.

## Introduction

Bipolar disorder affects about 1% to 2% of people worldwide and is marked by recurring periods of mania or hypomania separated by often lengthy and disabling depressive episodes [1]. Prognosis becomes even poorer when obsessive-compulsive disorder (OCD) is present, a combination found in roughly one-tenth to one-quarter of cases, which increases episode frequency and complicates treatment [2,3].

Relieving bipolar depression remains difficult. Core mood-stabilising agents such as lithium or valproate limit manic relapse but commonly leave residual depressive symptoms. Adding conventional antidepressants can help, yet carries a recognised risk of driving a manic or hypomanic switch; estimated switch rates range from roughly 20% to 40% in susceptible groups, even though more recent population work suggests slightly lower figures [4-6].

According to major guidelines (Canadian Network for Mood and Anxiety Treatments (CANMAT; 2023) [7], International Society for Bipolar Disorders (ISBD; 2016/2023) [5]), first-line pharmacological options for bipolar depression include quetiapine, lurasidone, lithium, lamotrigine, and the olanzapine-fluoxetine combination; conventional antidepressants are recommended only as adjuncts under mood-stabiliser cover because of switch risk. When residual depressive and obsessive symptoms persist despite optimised stabilisation, novel glutamate-targeted strategies are increasingly explored as low-switch alternatives to intravenous ketamine.

Attention has therefore shifted toward glutamate-focused strategies. Sub-anaesthetic intravenous ketamine demonstrates rapid antidepressant effects, often within hours, in bipolar depression and, when given alongside a mood stabiliser, shows a relatively low propensity to induce mania [8-10]. Practical barriers remain, however: intravenous delivery is costly, clinic-bound, and can provoke transient psychotomimetic reactions. These constraints have incited interest in oral regimens capable of replicating ketamine's synaptogenic cascade.

The Cheung Glutamatergic Regimen (CGR) is a proposal that combines everyday substances to target different steps in the cascade. For example, dextromethorphan briefly blocks NMDA receptors, piracetam increases AMPA throughput, L-glutamine replenishes presynaptic stores, and a CYP2D6 inhibitor prolongs dextromethorphan exposure [11]. Complementary genetic and computational studies suggest that bipolar disorder may reflect excessive synaptic pruning; interventions that rebuild glutamatergic connections could therefore stabilise circuits rather than merely raise excitability [12,13].

Some patients cannot tolerate pharmaceutical CYP2D6 inhibitors. Botanical alternatives such as goldenseal root extract, a moderate inhibitor, have been explored as substitutes [14]. The present report describes a case of bipolar I disorder with prominent obsessive-compulsive features in which short-term goldenseal augmentation of an oral dextromethorphan-piracetam-glutamine stack produced noticeable mood improvement without triggering hypomania. This case report illustrates a low-risk option for treatment-intolerant individuals and underlines the need for careful monitoring and controlled trials.

## Case presentation

The patient was a 32-year-old man who had been monitored in our clinic since July 2025 for bipolar I disorder with prominent obsessive-compulsive features and attentional deficits suggestive of comorbid attention-deficit/hyperactivity disorder (ADHD). The bipolar I diagnosis was supported by a clear manic episode in 2021 (lasting up to two weeks), characterised by racing ideas, impulsive buying, binge eating, elevated energy, and reckless driving of the family car, resulting in a motor-vehicle accident, constituting marked impairment. Shorter hypomanic bursts (one to two days to one week) have recurred intermittently since. 

When first assessed, he was in a severe depressive phase characterised by anxious rumination, prolonged sleep-onset latency, irritability, and marked reactivity to minor social stressors. He traced his illness back at least to 2021, describing one- to two-week bursts of hypomanic energy featuring racing ideas, impulsive spending, binge eating, and one motor-vehicle accident attributed to reckless driving. Lifelong difficulties with focus and task completion suggested comorbid ADHD, and intrusive, health-centred worries supported an OCD spectrum diagnosis. Formal schooling had ended during Form 4 because of academic failure. Previous drug treatment in 2021 had helped temporarily, but records were fragmentary. Previous treatment in 2021 included venlafaxine (Efexor) plus valproate for six months with partial benefit.

During the intake visit on 18 July 2025, his Patient Health Questionnaire-9 (PHQ-9) [15] was 21, and Generalized Anxiety Disorder-7 (GAD-7) [16] was 19. Guideline-concordant stabilisation was initiated with valproate 500 mg nightly (titrated to 800 mg total daily), a low-dose atypical antipsychotic (initially risperidone 0.5 mg, later switched to aripiprazole 2.5-5 mg), Deanxit, lemborexant, and alprazolam as needed (prn). Quetiapine was considered but not selected because of the patient’s binge-eating history and preference to avoid additional sedation and metabolic risk; lithium was discussed but declined owing to the monitoring burden and risks of toxicity. A short trial of bupropion XL 150 mg in August for residual apathy and suspected ADHD was discontinued within ten days because of jitteriness.

Mid-August ushered in an oral glutamatergic protocol designed to mimic ketamine's cascade: dextromethorphan 45 mg/day divided, piracetam 600-1 200 mg/day divided, and L-glutamine 1 000 mg/day. Mood-stabiliser coverage was maintained with valproate titrated to 800 mg, aripiprazole 2.5-5 mg, pregabalin 50-75 mg nightly, propranolol 20 mg, clonazepam 0.5 mg prn, and intermittent methylphenidate LA 10 mg for attentional lapses. Over September-December 2025, he noted fewer angry outbursts and improved tolerance of crowds; nonetheless, PHQ-9 values remained 12-15 and GAD-7 8-16, dipping when he missed dextromethorphan or piracetam doses.

In early January 2026, he sought further improvement without risking a hypomanic swing. We therefore added goldenseal root extract 600 mg daily (standardised for berberine/hydrastine) for 10 days to inhibit CYP2D6 moderately and prolong dextromethorphan exposure. The rest of the stack, dextromethorphan 45-90 mg/day, piracetam 1 200 mg/day, L-glutamine 2 000 mg/day, and the mood-stabiliser backbone were unchanged. During these 10 days, he reported a brighter mood, higher energy, and greater engagement with family duties, while neither reduced sleep need nor racing thoughts emerged.

Goldenseal was stopped after the planned course. By January 31, 2026, depressive affect had resurfaced (PHQ-9 = 18; GAD-7 = 13), though intrusive thoughts remained less oppressive and daily functioning was acceptable. As no gastrointestinal or hepatic adverse effects had occurred, goldenseal (600 mg daily) was reinstated with close monitoring.

**Figure 1:**
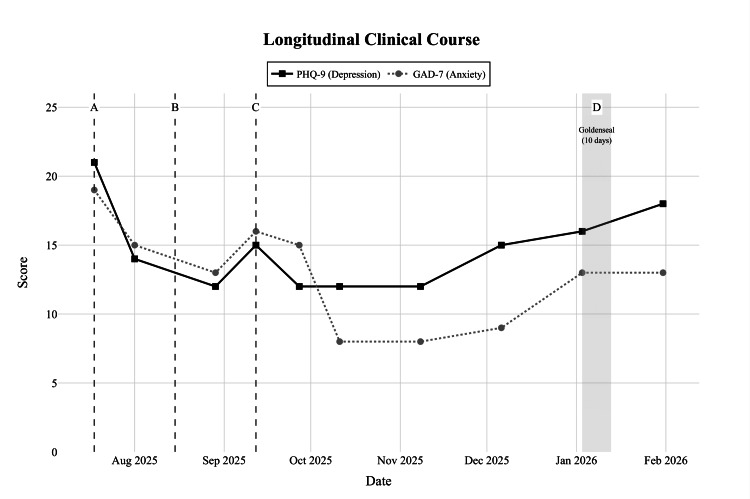
Timeline of PHQ-9 (depression) and GAD-7 (anxiety) scores over the course of treatment Vertical lines and shaded areas indicate key pharmacological changes. A: Initiation of valproate, aripiprazole, and Deanxit (July 2025); B: Addition of dextromethorphan and methylphenidate (Ritalin) (August 2025); C: Addition of piracetam (September 2025). D (Shaded): 10-day trial of Goldenseal (CYP2D6 inhibitor) in early January 2026. Note the transient improvement followed by a resurgence of symptoms (PHQ-9 = 18) recorded on 31 Jan 2026 after cessation. PHQ-9: Patient Health Questionnaire-9 ; GAD-7: Generalized Anxiety Disorder-7

This chronology (Figure 1) documents a brief yet meaningful antidepressant response to botanical CYP2D6 inhibition layered on an oral dextromethorphan-piracetam-glutamine regimen, achieved without precipitating hypomania in a patient whose bipolar depression is complicated by obsessive ruminations, health-related looping thoughts, crowd anxiety, and lifelong attentional/executive difficulties (evident since childhood school years).

## Discussion

In this patient, a 10-day course of the CGR enhanced with goldenseal produced a clear, albeit short-lived, antidepressant response. While taking dextromethorphan 45-90 mg, piracetam 1 200 mg, l-glutamine 2 000 mg, and botanical CYP2D6 inhibition (goldenseal 600 mg), he reported a brighter mood and greater day-to-day engagement, yet displayed no hypomanic warning signs despite a documented history of such episodes. Symptom resurgence within days of discontinuing goldenseal, followed by renewed benefit after re-exposure, strengthens the temporal link and supports the regimen's principal design: brief NMDA blockade paired with AMPA facilitation and glutamate replenishment to drive rapid synaptic plasticity [11].

Genetic data lend credence to this glutamatergic-repair model. A recent analysis of large bipolar disorder genome-wide association studies showed that microglia-mediated synaptic pruning pathways, not classical glutamatergic genes, dominate heritability signals and remain significant after controlling for glutamatergic overlap [12]. Transcriptome-wide results indicated excess expression of pruning activators, suggesting that over-elimination of synapses may leave cortico-limbic circuitry sparse and unstable. Mendelian randomisation further implied that greater cognitive reserve, often protective in major depression, exacerbates bipolar disorder risk, perhaps by allowing residual networks to overshoot into mania once stressors arise. On that reading, interventions that rebuild connections, rather than merely boost monoamines, should stabilise circuits without heightening manic liability.

Computational studies reinforce this view. A neural-network pruning model compared ketamine-like synaptogenic regrowth, selective serotonin reuptake inhibitor (SSRI)-like slow optimisation, and neurosteroid-like tonic inhibition [13]. Only the ketamine analogue reduced post-treatment relapse to near zero and eliminated manic-proxy activation, whereas the SSRI analogue carried the highest relapse and manic indices. The present case echoes those simulations: goldenseal-prolonged dextromethorphan produced benefit without a switch, contrasting with the patient's past intolerance of bupropion.

Clinically, scattered reports already describe rapid relief with oral CGR variants in refractory bipolar depression, OCD, and ADHD, provided a mood stabiliser remains in place [11]. Our observation extends this literature by demonstrating that a non-prescription botanical inhibitor, goldenseal, can substitute for pharmaceutical CYP2D6 blockers when these are poorly tolerated. Goldenseal's moderate inhibitory strength (≈40%-50% reduction in metabolic ratio) [14] appears sufficient to elevate dextromethorphan exposure for a discernible effect, and its short half-life allows swift withdrawal if activation emerges.

That said, goldenseal presents well-known problems. Alkaloid content varies widely across brands, oral absorption is erratic, and animal studies flag hepatotoxic and carcinogenic signals at sustained high doses [17]. It also inhibits CYP3A4 and P-glycoprotein, raising interaction concerns in polypharmacy. In our patient, no adverse events were observed, but the exposure was brief, and laboratory monitoring was limited.

Several limitations temper interpretation. First, this is a single-case observation. Valproate, aripiprazole, pregabalin, and Deanxit may all have contributed to mood trajectories, and spontaneous fluctuation or expectancy cannot be excluded. Second, no plasma dextromethorphan levels or CYP2D6 activity markers were taken, so the magnitude of inhibition is inferred, not proved. Third, the manic switch assessment relied on clinical interviews rather than structured rating scales. Finally, the patient's East Asian ancestry and comorbid ADHD/OCD limit generalisability.

Even so, the pattern fits a broader narrative: bipolar disorder may be driven less by neurotransmitter deficits and more by over-pruning of synapses, making circuit-restorative agents attractive. Brief, titratable interventions that promote synaptogenesis, whether intravenous ketamine or oral dextromethorphan plus AMPA modulators, seem to improve mood quickly without the switch liability that shadows monoaminergic antidepressants. Botanical CYP2D6 inhibitors could widen access where prescription agents are intolerable or unavailable, provided quality control and safety issues are addressed.

## Conclusions

Future work should move beyond anecdote. Randomised trials ought to compare pharmaceutical and botanical inhibitors within CGR, include pharmacokinetic sampling, and track manic symptoms longitudinally. Genomic-pruning markers could serve as predictors of response, and computational models might guide dose-finding. Until such data emerge, clinicians should reserve goldenseal-augmented regimens for carefully selected, mood-stabilised patients, with close monitoring for hepatic function, drug interactions, and emergent hypomania.

## References

[1] Merikangas KR, Jin R, He JP (2011). Prevalence and correlates of bipolar spectrum disorder in the world mental health survey initiative. Arch Gen Psychiatry.

[2] Amerio A, Odone A, Marchesi C, Ghaemi SN (2014). Treatment of comorbid bipolar disorder and obsessive-compulsive disorder: a systematic review. J Affect Disord.

[3] Ferentinos P, Preti A, Veroniki AA, Pitsalidis KG, Theofilidis AT, Antoniou A, Fountoulakis KN (2020). Comorbidity of obsessive-compulsive disorder in bipolar spectrum disorders: Systematic review and meta-analysis of its prevalence. J Affect Disord.

[4] Tondo L, Vázquez G, Baldessarini RJ (2010). Mania associated with antidepressant treatment: comprehensive meta-analytic review. Acta Psychiatr Scand.

[5] Pacchiarotti I, Bond DJ, Baldessarini RJ (2013). The International Society for Bipolar Disorders (ISBD) task force report on antidepressant use in bipolar disorders. Am J Psychiatry.

[6] Viktorin A, Lichtenstein P, Thase ME, Larsson H, Lundholm C, Magnusson PK, Landén M (2014). The risk of switch to mania in patients with bipolar disorder during treatment with an antidepressant alone and in combination with a mood stabilizer. Am J Psychiatry.

[7] Keramatian K, Chithra NK, Yatham LN (2023). The CANMAT and ISBD guidelines for the treatment of bipolar disorder: summary and a 2023 update of evidence. Focus (Am Psychiatr Publ).

[8] Sanacora G, Zarate CA, Krystal JH, Manji HK (2008). Targeting the glutamatergic system to develop novel, improved therapeutics for mood disorders. Nat Rev Drug Discov.

[9] Bahji A, Zarate CA, Vazquez GH (2021). Ketamine for bipolar depression: a systematic review. Int J Neuropsychopharmacol.

[10] Fancy F, Haikazian S, Johnson DE (2023). Ketamine for bipolar depression: an updated systematic review. Ther Adv Psychopharmacol.

[11] Cheung N (2025). Neuroplasticity in bipolar disorder with a ketamine-like glutamatergic regimen: a mechanistic review. Zenodo.

[12] Cheung N (2026). From pruned circuits to manic instability: genetic evidence for independent pruning dominance and risk-amplifying cognitive reserve in bipolar disorder. Zenodo.

[13] Cheung N (2026). Modeling antidepressant-induced manic switch and longitudinal relapse: a unified pruning framework highlights glutamatergics' disease-modifying potential. Zenodo.

[14] Gurley BJ, Swain A, Hubbard MA (2008). Clinical assessment of CYP2D6-mediated herb-drug interactions in humans: effects of milk thistle, black cohosh, goldenseal, kava kava, St. John's wort, and Echinacea. Mol Nutr Food Res.

[15] Kroenke K, Spitzer RL, Williams JB (2001). The PHQ-9: validity of a brief depression severity measure. J Gen Intern Med.

[16] Spitzer RL, Kroenke K, Williams JB, Löwe B (2006). A brief measure for assessing generalized anxiety disorder: the GAD-7. Arch Intern Med.

[17] Mandal SK, Maji AK, Mishra SK, Ishfaq PM, Devkota HP, Silva AS, Das N (2020). Goldenseal (Hydrastis canadensis L.) and its active constituents: a critical review of their efficacy and toxicological issues. Pharmacol Res.

